# *Staphylococcus aureus* Infection Initiates Hypoxia-Mediated Transforming Growth Factor-β1 Upregulation to Trigger Osteomyelitis

**DOI:** 10.1128/msystems.00380-22

**Published:** 2022-07-19

**Authors:** Wei Zhang, Yiwei Lin, Yang Zong, Xin Ma, Chaolai Jiang, Haojie Shan, Wenyang Xia, Lifu Yin, Nan Wang, Lihui Zhou, Zubin Zhou, Xiaowei Yu

**Affiliations:** a Department of Orthopaedic Surgery, Shanghai Jiao Tong University Affiliated Sixth People’s Hospital, Shanghai, China; b Department of Emergency, the First Affiliated Hospital of Zhengzhou University, Zhengzhou, Henan, China; c Department of Orthopaedic Surgery, Xiangshan First People’s Hospital, Ningbo, Zhejiang, China; Mayo Clinic

**Keywords:** *Staphylococcus aureus*, osteomyelitis, hypoxia, hypoxia-inducible factor-1α (HIF-1α), transforming growth factor-β1 (TGF-β1)

## Abstract

Little is unknown about the regulatory mechanisms underlying the pathogenesis of osteomyelitis induced by Staphylococcus aureus. Hypoxia-inducible factor-1α (HIF-1α) and transforming growth factor β1 (TGF-β1) were both upregulated in S. aureus-infected MC3T3-E1 cells and osteomyelitis patients. HIF-1α directly targets the hypoxia-responsive elements (HREs) of TGF-β1 mRNA to induce its expression. Silencing HIF-1α and TGF-β1, as well as treatment of hypoxia inhibitor IDF-11774, consistently elevated OPN and RUNX2 expression and alizarin Red S (ARS) and alkaline phosphatase (ALP) staining levels in MC3T3-E1 cells with S. aureus infection. S. aureus infection increased HIF-1α expression and serum TGF-β1 concentration in a mouse model of osteomyelitis. Hypoxia inhibitor IDF-11774 treatment reduced serum levels of interleukin (IL)-6, IL-1β, and C-reactive protein. Upon S. aureus infection, hypoxia was activated to trigger TGF-β1 upregulation through direct targeting of HRE on TGF-β1 mRNA by HIF-1α, eventually leading to osteomyelitis symptoms in terms of osteogenesis and mineralization deficiencies as well as elevated inflammation. This study hereby suggests a novel signaling cascade involving hypoxia/HIF-1α/TGF-β1 in osteomyelitis pathogenesis, which could potentially serve as a target for therapeutic measures.

**IMPORTANCE** The pathogenesis of osteomyelitis induced by Staphylococcus aureus remains unclear. To develop therapeutic approaches for osteomyelitis, it is important to understand the molecular mechanisms of its pathogenesis. Our results suggests that hypoxia/HIF-1α/TGF-β1 signaling is involved in osteomyelitis pathogenesis. Thus, these findings highlight the potential of this signaling components as therapeutic targets for the treatment of osteomyelitis.

## INTRODUCTION

In the orthopedic field, osteomyelitis is a drastic condition, a bone or bone marrow infection that leads to progressive inflammatory responses and damage to bone tissue (1). Staphylococcus aureus is a major cause of osteomyelitis worldwide (2). This infection is not only debilitating and painful, but also tends to be chronic, making treatment extremely challenging. Treatment for infectious osteomyelitis can also be difficult because the types of pathogenic organisms and their sensitivities to drugs greatly vary, which is worsened by increasing numbers of implant-associated infections, antibiotic-resistant bacterial strains, and elderly patients whose immune systems are compromised (3). This results in a high rate of clinical osteomyelitis treatment failures, often leading to amputation and/or loss of function (1). Even though some cases of failed treatment are accounted for by the accelerated spread of drug-resistant strains, including methicillin-resistant S. aureus (4), many therapy-insensitive cases of osteomyelitis are due to infections by strains that are not yet antibiotic resistant (5). Hence, the main determinants of ineffectiveness of antimicrobial treatments are most likely the interactions of staphylococci with osseous tissue and the various strategies employed by S. aureus to tame the host immune system (6).

To develop more effective therapeutic approaches, a more comprehensive understanding about the mechanism employed by S. aureus to elicit infections in bone and the complex pathogenesis of osteomyelitis is needed. The transforming growth factor-β (TGF-β) superfamily is an ancient metazoan protein class universally involved in cell and tissue differentiation, immunology, and developmental biology (7). In mammals, three TGF-β isoforms have been characterized, namely, TGF-β1, TGF-β2, and TGF-β3. Activated TGF-β1 is an essential cytokine that exhibits effects on inflammation-induced bone resorption disorders, including osteomyelitis, both *in vivo* and *in vitro* (8, 9).

On the other hand, hypoxia is associated with osteomyelitis (10), and chronic hypoxia has been long recognized as a systemic factor of osteomyelitis (11, 12). Hypoxia-inducible factor-1α (HIF-1α) is a transcriptional activator which mediates adaptive responses to hypoxic conditions. Under normal oxygen (normoxic) levels, the prolyl hydroxylase (PHD) hydroxylates HIF-1α (13), causing HIF-1α to be polyubiquitinated and degraded in the cytosol (14). Under hypoxic conditions, PHDs are suppressed, resulting in the stabilization of HIF-1α and their subsequent translocation into the nucleus, where they bind to hypoxia-responsive elements (HREs) to initiate transcription of targeted genes (15). Interestingly, hypoxia regulation of TGF-β1 has been implicated in several human diseases. For instance, hypoxia elicits TGF-β1 secretion in mesenchymal stem cells, which in turn promotes the progression of breast cancer (16). In dermal fibroblasts, HIF-1α reportedly activates the TGF-β1/Smad signaling and increases collagen deposition (17). Also, by activating the TGF-β1 pathways, HIF-1α could promote the development of keloid (18) and pulmonary fibrosis (19).

Given the reported correlation between hypoxia and TGF-β1, and more importantly, their involvement in osteomyelitis, we aimed to investigate their roles in S. aureus infection-inflicted osteomyelitis cell culture and animal models.

## RESULTS

### HIF-1α and TGF-β1 are upregulated in MC3T3-E1 cells infected with *S. aureus* and in osteomyelitis patients.

**FIG 1:**
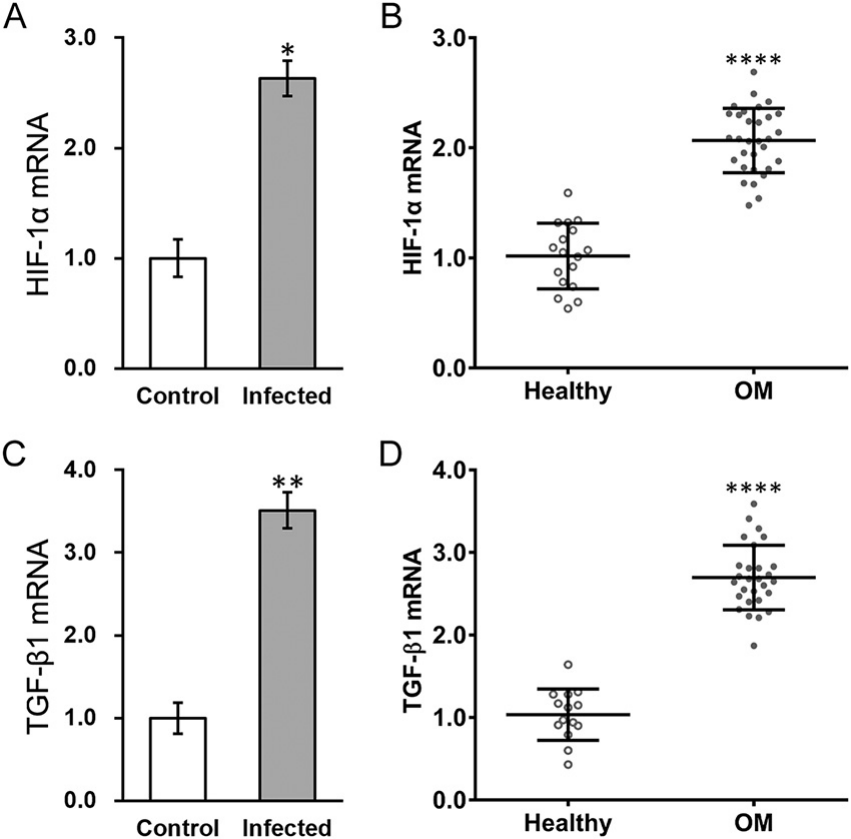
Hypoxia-inducible factor-1α (HIF-1α) and transforming growth factor β1 (TGF-β1) are upregulated in S. aureus-infected MC3T3-E1 cells and osteomyelitis patients. (A and B) Expression of HIF-1α mRNA in (A) control and S. aureus-infected MC3T3-E1 cells and in (B) healthy controls (*n* = 14) and osteomyelitis patients (OM, *n* = 28). (C and D) Expression of TGF-β1 mRNA in (C) control and S. aureus-infected MC3T3-E1 cells and in (D) healthy controls (*n* = 16) and osteomyelitis patients (OM, *n* = 30). Data are presented as mean ± standard deviation (SD) from three technical replicates. *, *P* < 0.05; **, *P* < 0.01; ****, *P* < 0.0001 for control versus infected and healthy versus OM, as determined by analysis of variance (ANOVA) test.

Hypoxia is reportedly associated with osteomyelitis (10), and chronic hypoxia has long been recognized as a systemic factor of osteomyelitis (11, 12). Therefore, we first measured the expression of a hypoxia indicator, HIF-1α, in MC3T3-E1 cells with S. aureus infection compared with that in control cells. As shown in Fig. 1A, there was a marked upregulation of HIF-1α mRNA in MC3T3-E1 cells at 7 days after infection with S. aureus. Consistent findings were revealed in clinical analysis involving 28 osteomyelitis patients, whose serum HIF-1α mRNA levels were also much higher than those of the 14 healthy controls with matched age, gender, body mass index (BMI), and demographic (Fig. 1B).

Next, we set out to identify the downstream effector of HIF-1α in the osteomyelitis setting. Because hypoxia regulation of TGF-β1 has been implicated in several human diseases (16–19), we examined its expression in MC3T3-E1 cells exposed to S. aureus in comparison to that in untreated cells. As shown in Fig. 1C, TGF-β1 mRNA was upregulated to a significant extent in infected cells. To consolidate this result in clinical samples, TGF-β1 mRNA was also upregulated in osteomyelitis patients (Fig. 1D). According to these results, HIF-1α and TGF-β1 were upregulated in S. aureus-exposed MC3T3-E1 cells and in osteomyelitis patients.

### HIF-1α directly targets the HRE of TGF-β1 mRNA to induce its expression in MC3T3-E1 cells.

**FIG 2:**
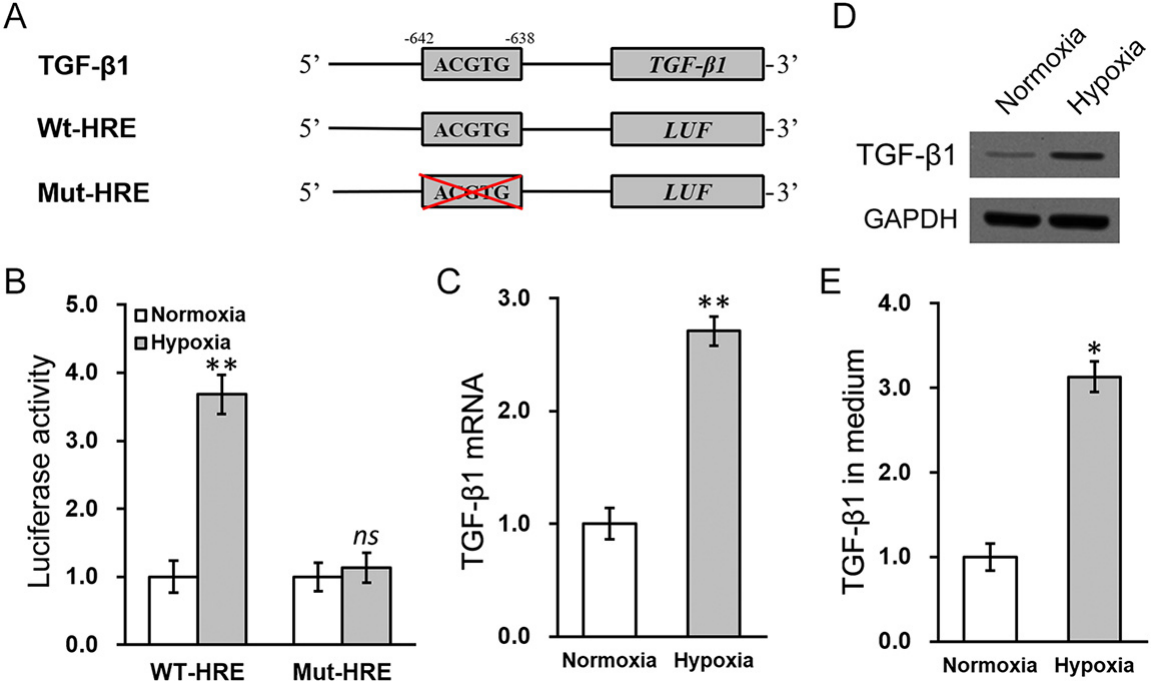
HIF-1α directly targets the hypoxia-responsive element (HRE) of TGF-β1 mRNA to induce its expression in MC3T3-E1 cells. (A) Promoter sequences of TGF-β1 mRNA containing the wild-type (WT) or mutated (Mut) HRE were cloned at the upstream of a luciferase reporter open reading frame (LUF). (B) Luciferase activities of WT-HRE and Mut-HRE constructs were measured in MC3T3-E1 cells cotransfected under either normoxic or hypoxic conditions. (C) Intracellular TGF-β1 mRNA, (D) intracellular TGF-β1 protein expression, and (E) TGF-β1 concentration in the culture medium were measured in MC3T3-E1 cells cotransfected under either normoxic or hypoxic conditions. Data are presented as mean ± SD from at least three independent experiments, each with three technical replicates. ns, *P* > 0.05; *, *P* < 0.05; **, *P* < 0.01 for normoxia versus hypoxia, as determined by ANOVA test.

The target genes of HIF-1α contain HREs (15), the majority of which consist of the core sequence ACGTG (20). Through genomic sequence analysis, this HRE sequence was identified in the promoter region of TGF-β1 mRNA (Fig. 2A). To verify this potential HRE, the promoter region of TGF-β1 mRNA, with the wild-type (Wt-HRE) or mutated HRE sequence (Mut-HRE), respectively, was cloned at the upstream region of a luciferase reporter open reading frame (LUF) (Fig. 2A). Under hypoxic conditions, the activity of Wt-HRE LUF was significantly increased compared to that under the normoxic condition, whereas the activity of Mut-HRE LUF was similar in both normoxic and hypoxic conditions (Fig. 2B). Indeed, the hypoxic condition could trigger significant upregulation of TGF-β1 at both transcript and protein levels in MC3T3-E1 cells (Fig. 2C and D), as well as its secretion into the culture medium (Fig. 2E). These data convincingly suggested that in MC3T3-E1 cells, HIF-1α could directly target the HRE of TGF-β1 mRNA to induce its expression.

### Silencing HIF-1α rescues defects in osteogenesis and mineralization in MC3T3-E1 cells infected with *S. aureus*.

**FIG 3:**
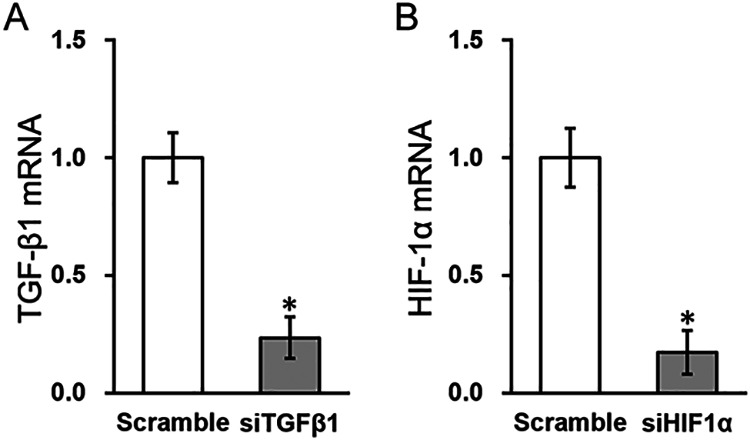
Effect of small interfering RNA (siRNA) silencing on TGF-β1 and HIF-1α expression in MC3T3-E1 cells. MC3T3-E1 cells were transfected with either scramble siRNA, (A) TGF-β1 siRNA (siTGFβ1), or (B) HIF-1α siRNA (siHIF1α), respectively. Data are presented as mean ± SD from at least three independent experiments, each with three technical replicates. *, *P* < 0.05 for scramble versus siTGFβ1 or siHIF1α, as determined by ANOVA test.

Next, to investigate the roles of HIF-1α and TGF-β1 in S. aureus infection-induced osteomyelitis, specific silencing of these two genes was introduced into MC3T3-E1 cells (see Materials and Methods). As shown in Fig. 3A and B, siTGFβ1 and siHIF1α caused marked inhibition of TGF-β1 and HIF-1α expression, respectively, compared to scramble small interfering RNA (siRNA) controls.

**FIG 4:**
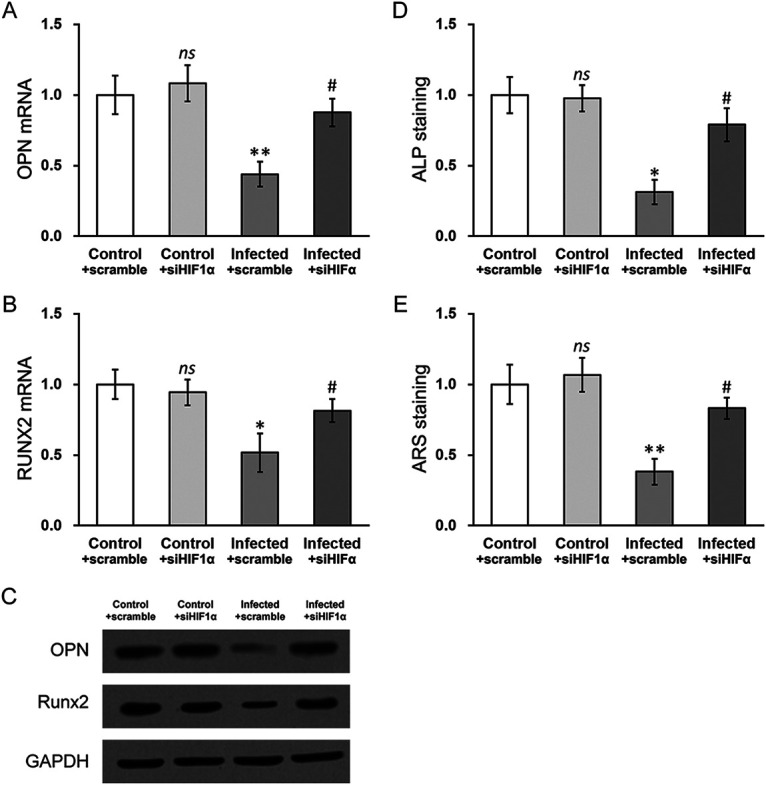
Silencing HIF-1α rescues defects in osteogenesis and mineralization in MC3T3-E1 cells with S. aureus infection. mRNA levels of osteogenic markers (A) OPN and (B) RUNX2, as well as their protein levels (C), were measured in control and S. aureus-infected MC3T3-E1 cells with either scramble siRNA or HIF-1α siRNA (siHIF1α). (D) Alkaline phosphatase (ALP) and (E) alizarin red S (ARS) staining assays were performed in control and S. aureus-infected MC3T3-E1 cells with either scramble siRNA or HIF-1α siRNA (siHIF1α). Data are presented as mean ± SD from at least three independent experiments, each with three technical replicates. ns, *P* > 0.05 for control + scramble versus control + siHIF1α; *, *P* < 0.05; ** and *P* < 0.01 for infected + scramble versus control + scramble; #, *P* < 0.05 for infected + siHIF1α versus infected + scramble, as determined by ANOVA test.

The HIF-1α-silenced MC3T3-E1 cells were then subjected to S. aureus infection, followed by examination of the two osteogenic markers OPN and RUNX2. S. aureus infection caused obvious reduction in OPN and RUNX2 mRNA levels (Fig. 4A and B, infected + scramble versus control + scramble), which was largely restored by HIF-1α silencing (Fig. 4A and B, infected + siHIF1α versus infected + scramble). The protein levels of both osteogenic markers followed the same trend as their mRNAs. A mineralization defect, assayed by alkaline phosphatase (ALP) and alizarin Red S (ARS) staining, was another outcome following S. aureus infection in MC3T3-E1 cells (Fig. 4D to F, infected + scramble versus control + scramble). Upon HIF-1α silencing, both ALP and ARS staining showed that mineralization was rescued to levels similar to those in uninfected control cells (Fig. 4D to F, infected + siHIF1α versus infected + scramble). Taken together, these experiments demonstrated that HIF-1α mediated S. aureus infection-induced osteogenesis and mineralization defects in MC3T3-E1 cells.

### Silencing TGF-β1 rescues defects in osteogenesis and mineralization in MC3T3-E1 cells infected with *S. aureus*.

**FIG 5:**
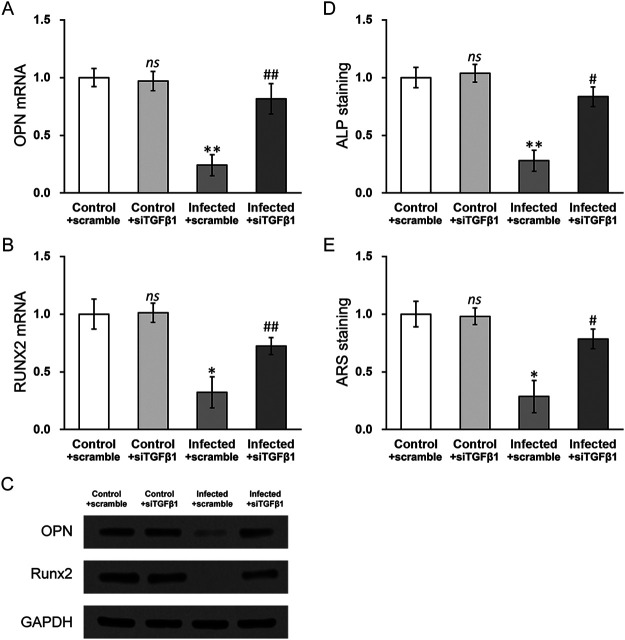
Silencing TGF-β1 rescues defects in osteogenesis and mineralization in MC3T3-E1 cells with S. aureus infection. mRNA levels of osteogenic markers (A) OPN and (B) RUNX2, as well as their protein levels (C), were measured in control and S. aureus-infected MC3T3-E1 cells with either scramble siRNA or TGF-β1 siRNA (siTGFβ1). (D) Alkaline phosphatase (ALP) and (E) alizarin Red S (ARS) staining assays were performed in control and S. aureus-infected MC3T3-E1 cells with either scramble siRNA or TGF-β1 siRNA (siTGFβ1). Data are presented as mean ± SD from at least three independent experiments, each with three technical replicates. ns, *P* > 0.05 for control + scramble versus control + siTGFβ1; *, *P* < 0.05 and **, *P* < 0.01 for infected + scramble versus control + scramble; #, *P* < 0.05 and ##, *P* < 0.01 for infected + siTGFβ1 versus infected + scramble, as determined by ANOVA test.

Likewise, TGF-β1-silenced MC3T3-E1 cells were also subjected to S. aureus infection, followed by examination of OPN and RUNX2 expression as well as mineralization assays (Fig. 5). As in the case of HIF-1α silencing, knocking down TGF-β1 resulted significantly restored expression of osteogenic markers OPN and RUNX2 (Fig. 5A to C) and greatly rescued mineralization in ALP and ARS stains (Fig. 5D to F), compared to that in the scramble siRNA control group (infected + siTGFβ1 versus infected + scramble). Therefore, these data indicated that TGF-β1 upregulation, likely caused by HIF-1α activation, is also responsible for the S. aureus-induced osteogenesis and mineralization defects in in MC3T3-E1 cells.

### Hypoxia inhibitor IDF-11774 rescues defects in osteogenesis and mineralization in MC3T3-E1 cells infected with *S. aureus*.

**FIG 6:**
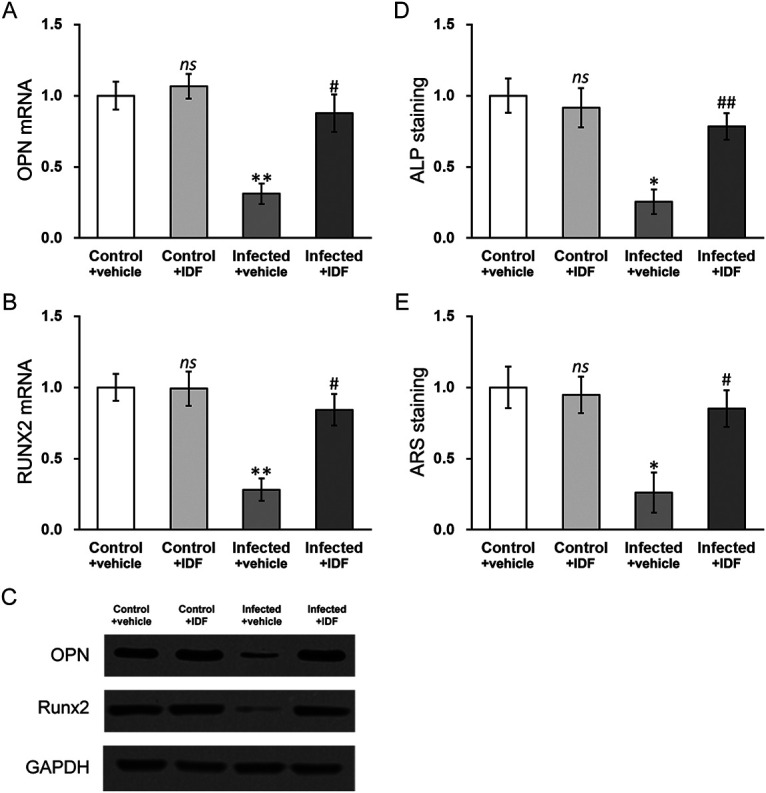
Hypoxia inhibitor IDF-11774 rescues defects in osteogenesis and mineralization in MC3T3-E1 cells with S. aureus infection. mRNA levels of osteogenic markers (A) OPN and (B) RUNX2, as well as their protein levels (C), were measured in control and S. aureus-infected MC3T3-E1 cells treated with either dimethyl sulfoxide (DMSO, vehicle) or 10 μM IDF-11774 (IDF). (D) Alkaline phosphatase (ALP) and (E) alizarin Red S (ARS) staining assays were performed in control and S. aureus-infected MC3T3-E1 cells treated with either DMSO (vehicle) or 10 μM IDF-11774 (IDF). Data are presented as mean ± SD from at least three independent experiments, each with three technical replicates. ns, *P* > 0.05 for control + vehicle versus control + IDF; *, *P* < 0.05 and **, *P* < 0.01 for infected + vehicle versus control + vehicle; #, *P* < 0.05 and ##, *P* < 0.01 for infected + IDF versus infected + vehicle, as determined by ANOVA test.

To provide additional support for the role of HIF-1α in S. aureus infection in MC3T3-E1 cells, we employed a novel hypoxia inhibitor called IDF-11774 (21) and tested its effect on osteogenesis and mineralization in MC3T3-E1 cells exposed to S. aureus (Fig. 6). As expected, 10 μM IDF-11774 (IDF) treatment significantly restored expression levels of RUNX2 and OPN, which were otherwise inhibited by S. aureus infection (Fig. 6A to C, infected + vehicle versus control + vehicle), compared to vehicle treatment (Fig. 6A to C, infected + IDF versus infected + vehicle). Moreover, S. aureus-induced mineralization defect, as evidenced by ALP and ARS assays, was also rescued by IDF-11774 treatment in MC3T3-E1 cells (Fig. 6D to F, infected + IDF versus infected + vehicle). These results, therefore, further confirmed the crucial role of hypoxia in S. aureus infection-induced osteogenesis and mineralization deficiencies.

### Hypoxia inhibitor IDF-11774 rescues serological parameters in mouse model of osteomyelitis.

**FIG 7:**
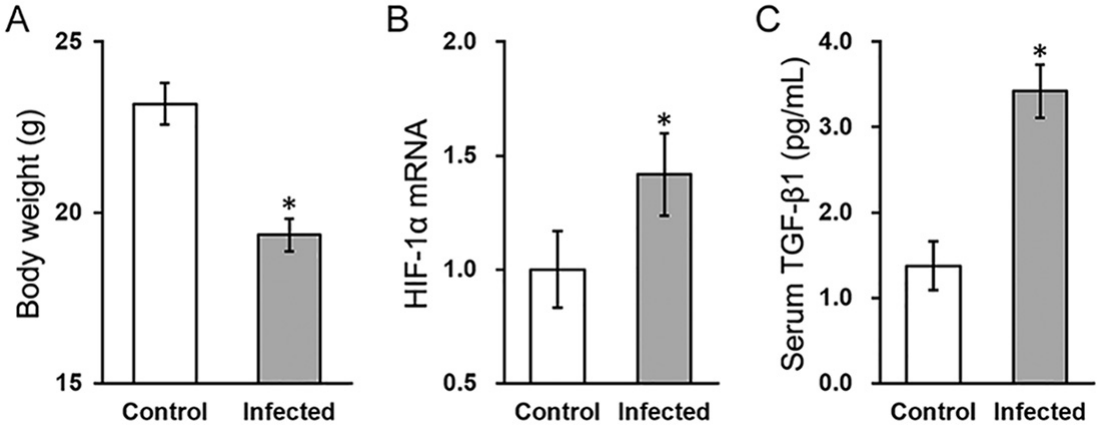
HIF-1α expression and serum TGF-β1 concentration are elevated in a mouse model of osteomyelitis. Mouse model of osteomyelitis was established (detailed in Materials and Methods), and on day 7 after S. aureus infection, (A) body weight, (B) blood HIF-1α mRNA expression, and (C) serum TGF-β1 concentration were measured. Data are presented as mean ± SD (*n* = 6 each group) from three technical replicates in each group. *, *P* < 0.05 for control versus infected, as determined by ANOVA test.

**FIG 8:**
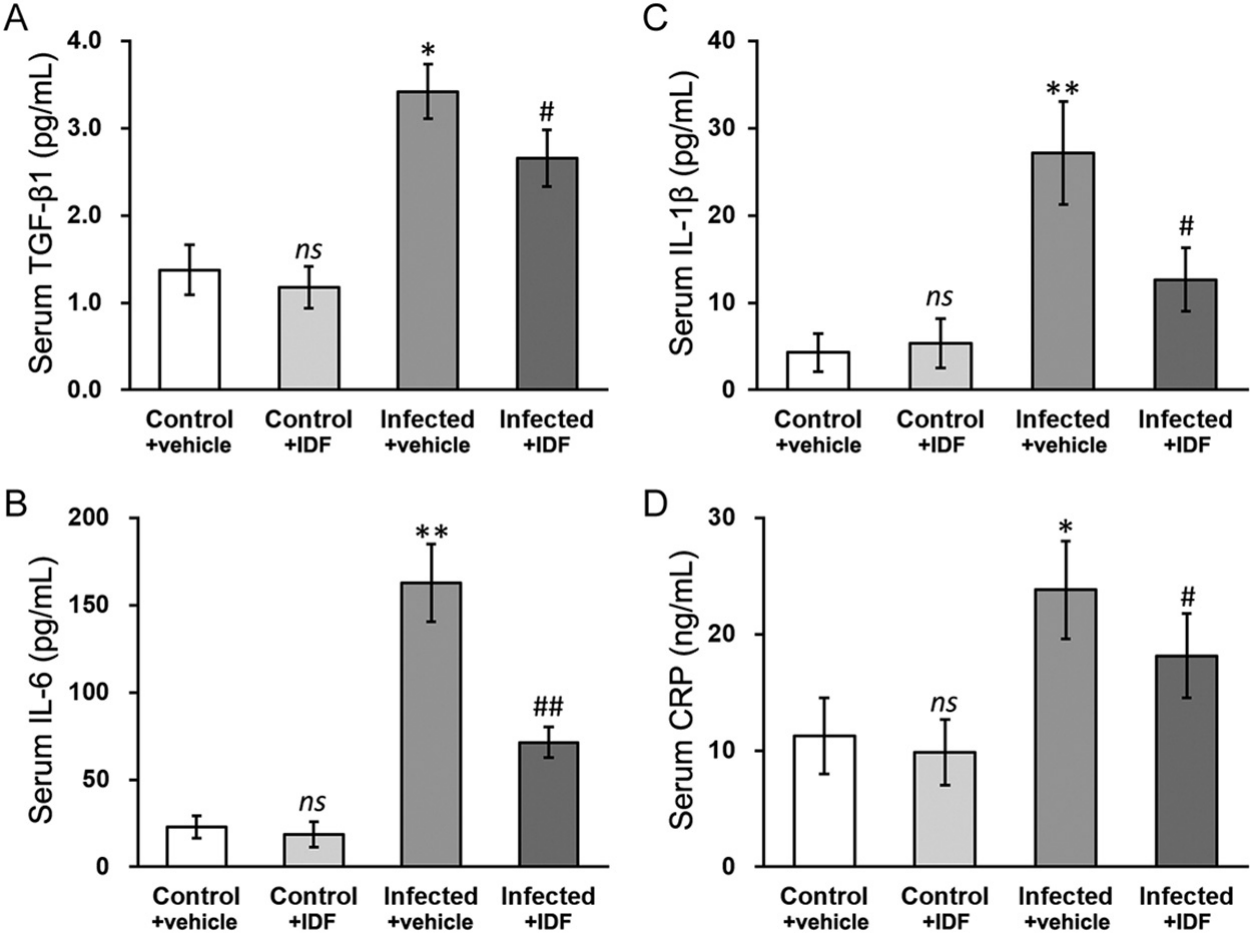
Hypoxia inhibitor IDF-11774 rescues serological parameters in mouse model of osteomyelitis. Mouse model of osteomyelitis was established and treated with either DMSO (vehicle) or 20 mg/kg/day IDF-11774 (IDF) (detailed in Materials and Methods). On day 7 after S. aureus infection, serum concentrations of (A) TGF-β1, (B) interleukin (IL)-6, (C) IL-1β, and (D) C-reactive protein (CRP) were measured. Data are presented as mean ± SD (*n* = 6 each group) from three technical replicates in each group. ns, *P* > 0.05 for control + vehicle versus control + IDF; *, *P* < 0.05 and **, *P* < 0.01 for infected + vehicle versus control + vehicle; #, *P* < 0.05 and ##, *P* < 0.01 for infected + IDF versus infected + vehicle, as determined by ANOVA test.

To further test the roles of HIF-1α and TGF-β1 in osteomyelitis *in vivo*, we employed a mouse model of S. aureus infection-induced osteomyelitis (22). At day 7 after initial inoculation, S. aureus infection caused significant reduction in body weight (Fig. 7), as well as elevated HIF-1α expression (Fig. 7B) and serum TGF-β1 concentration (Fig. 7C and 8A). Moreover, the serum levels of IL-1β and IL-6 in the infected group were increased compared to those in the control group on day 7 (Fig. 8B and C, infected + vehicle versus control + vehicle), which were moderately reduced by 20 mg/kg/day IDF-11774 treatment (Fig. 8B and C, infected + IDF versus infected + vehicle). Next, the serum level of C-reactive protein (CRP) was similarly increased by S. aureus infection (Fig. 8D, infected + vehicle versus control + vehicle), and was significantly reduced following IDF-11774 treatment (Fig. 8D, infected + IDF versus infected + vehicle).

## DISCUSSION

Osteomyelitis is a painful, debilitating, infectious disease of the bone marrow or bones that often results from S. aureus infection. In this investigation, we employed two models of S. aureus infection-induced osteomyelitis in order to reveal the roles of HIF-1α and TGF-β1 in its pathogenesis both *in vitro* and *in vivo*. MC3T3-E1 cells were employed in this work to assess osteoblast functions. Osteoblasts are the major type of functional cells involved in bone formation, and underlie the synthesis, secretion, and mineralization of the bone matrix. Our results demonstrated that S. aureus suppressed bone formation and mineralization. HIF-1α and TGF-β1 were upregulated during S. aureus infection in MC3T3-E1 cells, accompanied by strong inhibition of bone mineralization. In addition, expression levels of RUNX2 and OPN were shown to be downregulated in MC3T3-E1 cells exposed to S. aureus. Both RUNX2 and OPN are osteogenesis biomarkers, and their downregulation in MC3T3-E1 cells with S. aureus infection strongly indicated suppressed bone formation.

Thus, HIF-1α and TGF-β1 may be players in mineralization, bone formation, and osteogenesis, and siRNA silencing experiments were conducted to test this speculation. Indeed, knockdown of either HIF-1α or TGF-β1 in MC3T3-E1 cells exposed to S. aureus consistently restored expression of both OPN and RUNX2 and rescued bone mineralization, quantified as ARS and ALP staining levels. These data clearly demonstrated that HIF-1α and TGF-β1 upregulation were both critical in the S. aureus-induced osteogenesis and mineralization defects in MC3T3-E1 cells (Fig. 4 and 6). Combined with data shown in Fig. 2, where HIF-1α was shown to directly target the HRE of TGF-β1 mRNA to induce its expression in MC3T3-E1 cells, it can therefore be deduced that S. aureus infection causes hypoxia and subsequent upregulation of TGF-β1, which then results in deficiencies in osteogenesis and bone mineralization. This working model was further substantiated by the treatment using a hypoxia inhibitor, IDF-11774. Treatment with IDF-11774 closely mimicked the effect of silencing HIF-1α or TGF-β1 and rescued S. aureus infection-induced defects in MC3T3-E1 cells.

To further validate our *in vitro* results, we employed a commonly studied mouse model of S. aureus-induced osteomyelitis (22). IL-6 and IL-1β, mainly produced by activated monocytes/macrophages, have long been known to stimulate osteoclasts, resulting in bone resorption (23–25). Marriott et al. (26) using human bone tissues as well as a mouse model, showed that bone osteoblasts expressed IL-6 during bacterial infection. Yoshii et al. (27) similarly demonstrated that in a staphylococcal osteomyelitis model, local levels of IL-1β and IL-6 were increased in the infected bones. In this study, we also found that the serum concentrations of IL-1β and IL-6 in the infected group were notably higher. Moreover, the CRP level is a most valuable marker for assessing infectious processes during clinical practices (28). Consistent with these studies, in our mouse model, we also observed a high level of serum CRP following S. aureus infection, suggesting that our model can mimic the chronic inflammatory processes in osteomyelitis.

Having established the current osteomyelitis mouse model reproducing the infectious processes in humans, we proceeded to test whether the efficacy of the hypoxia inhibitor IDF-11774 in alleviating osteogenesis and mineralization defects *in vitro* could be recapitulated *in vivo*. To this end, we treated the S. aureus-infected mice with IDF-11774 and evaluated their serological parameters, including serum levels of IL-1β, IL-6, and CRP. We found that IDF-11774 administration to S. aureus-infected mice significantly repressed the upregulated serum levels of IL-6, IL-1β, and CRP, as well as TGF-β1. Considering the potent anti-hypoxia property of IDF-11774 (21), it can be postulated that the observed beneficial effects of IDF-11774 in S. aureus infection-inflicted serological phenotypes were mediated by inhibiting hypoxia/HIF-1α. In fact, inhibiting hypoxia by hyperbaric oxygen (HBO) therapy has long been hypothesized to reduce osteomyelitis symptoms (29). It has been reported that HBO enhanced wound healing in patients with chronic osteomyelitis (30). In line with these findings, our current study has not only raised a novel anti-hypoxia agent, IDF-11774, with potential against osteomyelitis, but also provided further theoretical basis in support of HBO therapy to treat osteomyelitis.

Besides hypoxia/HIF-1α, the results of this study propose TGF-β1 as a potential therapeutic target of osteomyelitis. In an earlier study, TGF-β1 activation exhibited important functions in scar formation in osteomyelitis, likely through the production of collagen I (31). In the same study, key members of the TGF-β1/Smad signaling pathway were implicated in a murine osteomyelitis model, but no information was uncovered concerning the factors responsible for activating TGF-β1 in the osteomyelitis setting. Here, have investigated in a similar osteomyelitis mouse model and revealed hypoxia/HIF-1α as the probable upstream activator of TGF-β1, thereby deepening the current understanding of osteomyelitis pathogenesis.

### Conclusion.

In conclusion, upon S. aureus infection, hypoxia is activated to trigger TGF-β1 upregulation through direct targeting of HRE on TGF-β1 mRNA by HIF-1α. Upregulated TGF-β1 leads to osteomyelitis symptoms, including osteogenesis and mineralization deficiencies and elevated inflammation. Our study has hereby proposed a novel signaling cascade involving hypoxia/HIF-1α/TGF-β1 in osteomyelitis pathogenesis, the components of which could potentially serve as targets for therapeutic measures. Nevertheless, our current study only involved a small sample pool with an *in vivo* animal model; therefore, more investigations are needed to further verify the results on a larger scale. In addition, given the observed alleviating effect of the hypoxia inhibitor IDF-11774, further investigations are warranted to potentiate its clinical efficacy in treating osteomyelitis.

## MATERIALS AND METHODS

### Cell line and *S. aureus* infection.

Mouse calvaria-derived osteo-precursor cells MC3T3-E1, acquired from American Type Culture Collection (ATCC, Rockville, MD, USA), were cultured under 5% CO_2_ at 37°C in minimum essential medium (alpha-MEM; Gibco, Grand Island, NY) with supplementation of 10% fetal bovine serum (FBS, Gibco), a 1% antibiotic cocktail (penicillin G sodium, streptomycin sulfate; Sigma-Aldrich), and 1% l-glutamine (Sigma-Aldrich). Every 48 h, the medium was replaced. When cells reached confluence, they were treated with trypsin, and only cells at passages 12 to 15 were utilized. S. aureus strain 6850 (53657; ATCC, Manassas, VA) was cultivated in BBL Trypticase Soy Broth (TSB; BD Biosciences, Franklin Lakes, NJ) overnight at 37°C under constant shaking, and then incubated at 37°C for 3 h to achieve exponential growth. Following incubation, tubes were centrifuged at 3,000 rpm for 10 min and the supernatant was then discarded. The remaining pellets were washed three times using phosphate-buffered saline (PBS) and resuspended in PBS until reaching a McFarland standard of 6. CFU per milliliter were verified using the spread plate method.

S. aureus infection in MC3T3-E1 cells was carried out based on established methods (32). Briefly, S. aureus culture was used to infect MC3T3-E1 cells at a multiplicity of infection of 100. After 21 days of incubation at 37°C, MC3T3-E1 cells were washed with PBS and treated with 20 mg/mL lysostaphin for 30 min to remove bacteria attached outside the cells, followed by the addition of fresh medium to the MC3T3-E1 cells.

### Patients.

We recruited 28 patients with confirmed diagnosis of S. aureus-infected osteomyelitis, according to positive bacteremia results in a blood culture, surgical sample, or percutaneous puncture aspiration. Fourteen healthy volunteers with matched age, gender, and BMI (data not shown) were also recruited. Blood samples were collected from all recruited participants and frozen at –80°C for further analysis. Written informed consent forms were acquired from all participants before collection of blood samples. This investigation obtained approval from the Ethics Committee of Shanghai Jiao Tong University Affiliated Sixth People’s Hospital (DWLL2018-0093). This study was performed in strict accordance with the Declaration of Helsinki Ethical Principles for Medical Research Involving Human Subjects.

### Quantitative real-time PCR.

Total mRNA was prepared using TRIzol (Invitrogen, Carlsbad, CA), and subsequently subjected to reverse transcription to generate cDNAs using Superscript II following the provided protocols (Bio-Rad, Hercules, CA). PCRs with triplicates from each sample were performed using the SYBR Green-based method (Applied Biosystems, Waltham, MA) with steps including 40 cycles of 95°C for 15 s and 60°C for 1 min. Relative expression levels were calculated with normalization to GAPDH using the threshold cycle (2^–ΔΔCT^) method. The primers used in this study are listed below (from 5′ to 3′):

Human *HIF-1α*, forward CATAAAGTCTGCAACATGGAAGGT, reverse ATTTGATGGGTGAGGAATGGGTT;

Human *TGF-β1*, forward CCCAGCATCTGCAAAGCTC, reverse GTCAATGTACAGCTGCCGCA;

Human *GAPDH*, forward CATCACTGCCACCCAGAAGACTG, reverse ATGCCAGTGAGCTTCCCGTTCAG;

Mouse *HIF-1α*, forward TGATGTGGGTGCTGGTGTC, reverse TTGTGTTGGGGCAGTACTG;

Mouse *TGF-β1*, forward ATACGTCAGACATTCGGGAAGCAG, reverse AATAGTTGGTATCCAGGGCTCTCCG;

Mouse *OPN*, forward CTTTCACTCCAATCGTCCCTAC, reverse GCTCTCTTTGGAATGCTCAAGT;

Mouse *RUNX2*, forward CGACAGTCCCAACTTCCTGT, reverse CGGTAACCACAGTCCCATCT;

Mouse *GAPDH*, forward CATCTCCTCCCGTTCTGCC, reverse GTGGTG-CAGGATGCATTGC.

### Luciferase reporter assay.

The promoter region of TGF-β1 containing the putative hypoxia response element was sub-cloned into the pGL3 luciferase reporter plasmid. Transient-transfection was performed with Lipofectamine 2000. The recipient cells were then subjected to either hypoxic (100 μM CoCl_2_) or normoxic (0 μM CoCl_2_) conditions for 24 h after transfection. The relative activities of luciferase were evaluated 24 h after the transfection using a commercial Bright-Glo Luciferase Reporter System (Promega, Madison, WI).

### Western blotting.

Cells were resuspended in lysis buffer consisting of 150 mM NaCl, 0.5 mM NaF, 50 mM Tris-HCl, 1 mM Na_3_VO_4_, 0.25% Na-deoxycholate, 0.1% NP-40 alternative, Protease Inhibitor Cocktail (1 tablet/10 mL, Roche), and 10 mM HEPES, with the pH adjusted to 7.4. Cell lysate samples were quantitatively evaluated using a bicinchoninic acid protein assay, and total protein (30 μg) was resolved through SDS-PAGE and then transferred to polyvinylidene difluoride membranes, which were subsequently blocked using 1% bovine serum albumin (BSA; Sigma-Aldrich) and incubated at 4°C overnight with the appropriate primary antibody. Primary antibodies for TGF-β1, OPN, RUNX2, and GAPDH (glyceraldehyde-3-phosphate dehydrogenase) were purchased from Abcam. Horseradish peroxidase-conjugated secondary antibodies were then utilized to visualize blots on an enhanced chemiluminescence-based imaging system.

### Small interfering RNA.

The siRNA experiments were performed with HIF-1α siRNA (sc-35561), TGF-β1 siRNA (sc-37192), and scramble control siRNA (sc-37007) following the protocol provided by Santa Cruz Biotechnology.

### Alkaline phosphatase staining assay.

Eight days following the establishment of osteogenesis, cells were washed twice with TB buffer (150 mM NaCl, 20 mM Tris, pH adjusted to 7.5,) and lysed using 100 μL lysis buffer (TB buffer, plus 0.1% Triton). Cell lysates were centrifuged at 4°C for 30 min at 12,000 rpm, and then the supernatants (45 μL) were mixed with 100 μL ALP substrate p-nitrophenyl phosphate liquid substrate system (Promega, Madison, WI, USA) for 20 min at 37°C. The absorbance was examined at 405 nm on a 96-well plate reader following the provided protocol. The ALP activity level was calculated and normalized to the amount of total protein.

### Alizarin Red-sulfate staining assay.

Eight days following the establishment of osteogenesis, an ARS staining assay with an osteogenesis assay kit (Millipore, Billerica, MA) was employed to evaluate mineralization. In brief, cells were fixed using formalin for alizarin Red S staining. The dye was later extracted from stained cells using 10% acetic acid and absorbance at 405 nm was measured. All data were expressed after normalization to the control values.

### HIF-1α inhibitor IDF-11774.

IDF-11774 (S8771, purity > 99%; Selleckchem, Houston, TX) was prepared as stock solution in dimethyl sulfoxide (DMSO) at 10 mM and stored at –20°C. In brief, 10 μM IDF-11774 was supplemented in cell culture, while 20 mg/kg/day IDF-11774 was administered orally to mice, according to previous methods (21).

### Mouse osteomyelitis model.

A mouse model of S. aureus infection-induced osteomyelitis was employed according to previously reported methods (22). Briefly, pathogen-free C57BL/6 12 weeks old male mice were purchased from the Animal Facility of the Shanghai Laboratory Animal Center and housed in accordance with institutional guidelines in cages with proper ventilation and *ad libitum* access to food and water. All experimental protocols were approved by the Ethical Board of Shanghai Jiao Tong University Affiliated Sixth People’s Hospital. Anesthetization of the animals was achieved using an intraperitoneal (i.p.) injection of pentobarbital (50 mg per kg body weight). The hair on the left knee was then shaved and the skin cleaned using povidone iodine. An incision was made over the left knee, and a lateral parapatellar arthrotomy was performed to medically replace the quadriceps-patellar complex and expose the distal femur. The distal end of the femur was penetrated with a 0.5-mm sharp steel burr on a high-speed drill (Fine Science Tools, Foster City, CA). Next, a hole was generated with a 23-gauge needle (outside diameter = 0.6 mm), through which 1 μL medium containing 1.0 × 10^8^ CFU S. aureus was inoculated via a Hamilton syringe into the medullary cavity of the femur. The control group was given PBS using the same protocol. Bone wax was used to seal the burr hole, the dislocated patella was restored, and the incisions of the skin/muscle were sutured closed. The animals were then left on a heating pad to recover under careful monitoring. Spontaneous forelimb movements and water intake were regarded as signs that the mice had recovered from anesthesia. Blood samples were collected from the mice by retro-orbital bleeding on day 7 after the infection.

### Enzyme-linked immunosorbent assay.

TGF-β1, IL-6, IL-1β, and CRP concentrations in the medium or serum were assessed with enzyme-linked immunosorbent assay kits specifically targeting those proteins (R&D Systems, Minneapolis, MN) following the provided protocols.

### Statistical analysis.

Data analyses were performed using GraphPad Prism (GraphPad software version 5, La Jolla, CA), and data are presented as means ± standard deviation (SD) unless otherwise noted. Differences between groups were determined by a one-way analysis of variance (ANOVA) test. *P* values of less than 0.05 were regarded as indications of statistical significance.

### Data availability.

The data that support the findings of this study are available from the corresponding author, Xiaowei Yu, upon reasonable request.

## References

[1] Lew DP, Waldvogel FA. 2004. Osteomyelitis. Lancet 364:369–379. doi:10.1016/S0140-6736(04)16727-5. 15276398

[2] Wright JA, Nair SP. 2010. Interaction of staphylococci with bone. Int J Med Microbiol 300:193–204. doi:10.1016/j.ijmm.2009.10.003. 19889575 PMC2814006

[3] Kurtz SM, Ong KL, Schmier J, Mowat F, Saleh K, Dybvik E, Kärrholm J, Garellick G, Havelin LI, Furnes O, Malchau H, Lau E. 2007. Future clinical and economic impact of revision total hip and knee arthroplasty. J Bone Joint Surg Am 89 Suppl 3:144–151. doi:10.2106/JBJS.G.00587. 17908880

[4] Chambers HF. 2005. Community-associated MRSA: resistance and virulence converge. N Engl J Med 352:1485–1487. doi:10.1056/NEJMe058023. 15814886

[5] Sheehy SH, Atkins BA, Bejon P, Byren I, Wyllie D, Athanasou NA, Berendt AR, McNally MA. 2010. The microbiology of chronic osteomyelitis: prevalence of resistance to common empirical anti-microbial regimens. J Infect 60:338–343. doi:10.1016/j.jinf.2010.03.006. 20230854

[6] Foster TJ. 2005. Immune evasion by staphylococci. Nat Rev Microbiol 3:948–958. doi:10.1038/nrmicro1289. 16322743

[7] Johnston CJ, Smyth DJ, Dresser DW, Maizels RM. 2016. TGF-beta in tolerance, development and regulation of immunity. Cell Immunol 299:14–22. doi:10.1016/j.cellimm.2015.10.006. 26617281 PMC4711336

[8] Lerner UH. 1994. Regulation of bone metabolism by the kallikrein-kinin system, the coagulation cascade, and the acute-phase reactants. Oral Surg Oral Med Oral Pathol 78:481–493. doi:10.1016/0030-4220(94)90043-4. 7528372

[9] Hausman MR, Rinker BD. 2004. Intractable wounds and infections: the role of impaired vascularity and advanced surgical methods for treatment. Am J Surg 187:44S–55S. doi:10.1016/S0002-9610(03)00304-0. 15147992

[10] Javed F, Correa FO, Nooh N, Almas K, Romanos GE, Al-Hezaimi K. 2013. Orofacial manifestations in patients with sickle cell disease. Am J Med Sci 345:234–237. doi:10.1097/MAJ.0b013e318265b146. 22990048

[11] Carek PJ, Dickerson LM, Sack JL. 2001. Diagnosis and management of osteomyelitis. Am Fam Physician 63:2413–2420. 11430456

[12] Lima AL, Oliveira PR, Carvalho VC, Cimerman S, Savio E, Diretrizes Panamericanas para el Tratamiento de las Osteomielitis e Infecciones de Tejidos Blandos Group. 2014. Recommendations for the treatment of osteomyelitis. Braz J Infect Dis 18:526–534. doi:10.1016/j.bjid.2013.12.005. 24698709 PMC9428226

[13] Ivan M, Haberberger T, Gervasi DC, Michelson KS, Günzler V, Kondo K, Yang H, Sorokina I, Conaway RC, Conaway JW, Kaelin WG. 2002. Biochemical purification and pharmacological inhibition of a mammalian prolyl hydroxylase acting on hypoxia-inducible factor. Proc Natl Acad Sci USA 99:13459–13464. doi:10.1073/pnas.192342099. 12351678 PMC129695

[14] Tanimoto K, Makino Y, Pereira T, Poellinger L. 2000. Mechanism of regulation of the hypoxia-inducible factor-1 alpha by the von Hippel-Lindau tumor suppressor protein. EMBO J 19:4298–4309. doi:10.1093/emboj/19.16.4298. 10944113 PMC302039

[15] Schodel J, Oikonomopoulos S, Ragoussis J, Pugh CW, Ratcliffe PJ, Mole DR. 2011. High-resolution genome-wide mapping of HIF-binding sites by ChIP-seq. Blood 117:e207–e217. doi:10.1182/blood-2010-10-314427. 21447827 PMC3374576

[16] Hung SP, Yang MH, Tseng KF, Lee OK. 2013. Hypoxia-induced secretion of TGF-β1 in mesenchymal stem cell promotes breast cancer cell progression. Cell Transplant 22:1869–1882. doi:10.3727/096368912X657954. 23067574

[17] Mingyuan X, Qianqian P, Shengquan X, Chenyi Y, Rui L, Yichen S, Jinghong X. 2018. Hypoxia-inducible factor-1α activates transforming growth factor-β1/Smad signaling and increases collagen deposition in dermal fibroblasts. Oncotarget 9:3188–3197. doi:10.18632/oncotarget.23225. 29423039 PMC5790456

[18] Lei R, Li J, Liu F, Li W, Zhang S, Wang Y, Chu X, Xu J. 2019. HIF-1α promotes the keloid development through the activation of TGF-β/Smad and TLR4/MyD88/NF-κB pathways. Cell Cycle 18:3239–3250. doi:10.1080/15384101.2019.1670508. 31645185 PMC6927730

[19] Lecarpentier Y, Gourrier E, Gobert V, Vallee A. 2019. Bronchopulmonary dysplasia: crosstalk between PPARγ, WNT/β-catenin and TGF-β pathways; the potential therapeutic role of PPARγ agonists. Front Pediatr 7:176. doi:10.3389/fped.2019.00176. 31131268 PMC6509750

[20] Wenger RH, Gassmann M. 1997. Oxygen(es) and the hypoxia-inducible factor-1. Biol Chem 378:609–616. 9278140

[21] Ban HS, Kim B-K, Lee H, Kim HM, Harmalkar D, Nam M, Park S-K, Lee K, Park J-T, Kim I, Lee K, Hwang G-S, Won M. 2017. The novel hypoxia-inducible factor-1α inhibitor IDF-11774 regulates cancer metabolism, thereby suppressing tumor growth. Cell Death Dis 8:e2843. doi:10.1038/cddis.2017.235. 28569777 PMC5520894

[22] Horst SA, Hoerr V, Beineke A, Kreis C, Tuchscherr L, Kalinka J, Lehne S, Schleicher I, Köhler G, Fuchs T, Raschke MJ, Rohde M, Peters G, Faber C, Löffler B, Medina E. 2012. A novel mouse model of *Staphylococcus aureus* chronic osteomyelitis that closely mimics the human infection: an integrated view of disease pathogenesis. Am J Pathol 181:1206–1214. doi:10.1016/j.ajpath.2012.07.005. 22902429

[23] Nishihara T, Ishihara Y, Noguchi T, Koga T. 1989. Membrane IL-1 induces bone resorption in organ culture. J Immunol 143:1881–1886. 2789251

[24] Ishimi Y, Miyaura C, Jin CH, Akatsu T, Abe E, Nakamura Y, Yamaguchi A, Yoshiki S, Matsuda T, Hirano T. 1990. IL-6 is produced by osteoblasts and induces bone resorption. J Immunol 145:3297–3303. 2121824

[25] Fu C, Shi R. 2020. Osteoclast biology in bone resorption: a review. STEMedicine 1:e57. doi:10.37175/stemedicine.v1i4.57.

[26] Marriott I, Gray DL, Tranguch SL, Fowler VG, Stryjewski M, Scott Levin L, Hudson MC, Bost KL. 2004. Osteoblasts express the inflammatory cytokine interleukin-6 in a murine model of *Staphylococcus aureus* osteomyelitis and infected human bone tissue. Am J Pathol 164:1399–1406. doi:10.1016/S0002-9440(10)63226-9. 15039227 PMC1615361

[27] Yoshii T, Magara S, Miyai D, Nishimura H, Kuroki E, Furudoi S, Komori T, Ohbayashi C. 2002. Local levels of interleukin-1β, -4, -6 and tumor necrosis factor alpha in an experimental model of murine osteomyelitis due to *Staphylococcus aureus*. Cytokine 19:59–65. doi:10.1006/cyto.2002.1039. 12182840

[28] Khan MH, Smith PN, Rao N, Donaldson WF. 2006. Serum C-reactive protein levels correlate with clinical response in patients treated with antibiotics for wound infections after spinal surgery. Spine J 6:311–315. doi:10.1016/j.spinee.2005.07.006. 16651226

[29] Calhoun JH, Cobos JA, Mader JT. 1991. Does hyperbaric oxygen have a place in the treatment of osteomyelitis? Orthop Clin North Am 22:467–471. doi:10.1016/S0030-5898(20)31675-8. 1852423

[30] Al-Waili NS, Butler GJ, Beale J, Abdullah MS, Hamilton RWB, Lee BY, Lucus P, Allen MWW, Petrillo RL, Carrey Z, Finkelstein M. 2005. Hyperbaric oxygen in the treatment of patients with cerebral stroke, brain trauma, and neurologic disease. Adv Ther 22:659–678. doi:10.1007/BF02849960. 16510383

[31] Wang P, Liu X, Xu P, Lu J, Wang R, Mu W. 2016. Decorin reduces hypertrophic scarring through inhibition of the TGF-β1/Smad signaling pathway in a rat osteomyelitis model. Exp Ther Med 12:2102–2108. doi:10.3892/etm.2016.3591. 27698699 PMC5038452

[32] Tuchscherr L, Medina E, Hussain M, Völker W, Heitmann V, Niemann S, Holzinger D, Roth J, Proctor RA, Becker K, Peters G, Löffler B. 2011. *Staphylococcus aureus* phenotype switching: an effective bacterial strategy to escape host immune response and establish a chronic infection. EMBO Mol Med 3:129–141. doi:10.1002/emmm.201000115. 21268281 PMC3395110

